# Combining stereotactic body radiotherapy with immunotherapy in stage IV non-small cell lung cancer

**DOI:** 10.3389/fonc.2023.1211815

**Published:** 2023-09-06

**Authors:** Xiaoli Liu, Alexander Chi

**Affiliations:** ^1^ Department of Radiation Oncology, Capital Medical University Xuanwu Hospital, Beijing, China; ^2^ School of Basic Medical Sciences, Capital Medical University, Beijing, China

**Keywords:** non-small cell lung cancer, metastasis, stereotactic body radiotherapy, immunotherapy, immune checkpoint inhibitors, abscopal effect

## Abstract

Immunotherapy has revolutionized the treatment of metastatic non-small cell lung cancer (NSCLC). Oligometastasis has been associated with better prognosis than widespread metastatic disease and may be curable by stereotactic body radiotherapy (SBRT). SBRT can stimulate immunogenic anti-tumor activity, which can be further augmented when combined with immunotherapy, such as immune checkpoint inhibitors (ICIs). Thus, its combination with immunotherapy was recognized as a promising treatment option, especially in the metastatic setting. However, the most optimal approach to combine SBRT with immunotherapy remains controversial with early clinical evidence emerging. Here, we review the current clinical evidence supporting the combination of SBRT with immunotherapy in the treatment of metastatic NSCLC. Also, we discuss the current controversies and areas for further exploration associated with this treatment strategy.

## Introduction

Lung cancer is the top cause of cancer-related deaths in the US and more than half of patients are diagnosed with metastatic disease, resulting in high mortality rates (1). Non-small cell lung cancer (NSCLC), the most common type of lung cancer, accounts for approximately 84% of all lung cancers (2). Immunotherapy, particularly immune checkpoint inhibitors (ICIs), has improved the survival of patients with metastatic NSCLC. However, response rates to ICIs remain suboptimal (3). SBRT, a form of precise radiotherapy, delivers high doses of radiation to the tumor target(s) with minimal side effects, and has been reported to induce immunogenic cell death (4, 5). Combining ICIs with local therapies like stereotactic body radiotherapy (SBRT) has shown promise in enhancing treatment efficacy both locally and distantly in areas outside of the irradiated area. Such distant effect is called the which abscopal effect. Such synergy between SBRT and ICIs, especially the combined treatment approach’s association with increased frequency of an abscopal response warrants further exploration and validation in large randomized controlled trials (RCTs) (6). Here, we provide an overview of the latest developments and challenges in combining SBRT with immunotherapy for stage IV NSCLC.

## Oligometastases in advanced non-small cell lung cancer: a systematic classification

The term “oligometastases” refers to an intermediate state between local disease and widespread metastases (7). Oligometastatic disease (OMD) is a state where “cure” or “long-time disease control” can be achieved through radical treatment (7, 8). Radiotherapy, surgery, and radiofrequency ablation have made previously untreatable lesions safer and more effective to treat, which prompted changes the definition of OMD (8). In 2019, the International Association for the Study of Lung Cancer (IASLC) proposed a consensus report that defined synchronous oligometastatic NSCLC as the presence of 1-5 distant metastases in a maximum of 3 organs (8). In 2020, the European Society for Radiotherapy and Oncology (ESTRO) and American Society for Radiation Oncology (ASTRO) reached a consensus on the definition of OMD, which is now defined as 1-5 metastatic lesions that can be safely treated (9).

**Figure 1:**
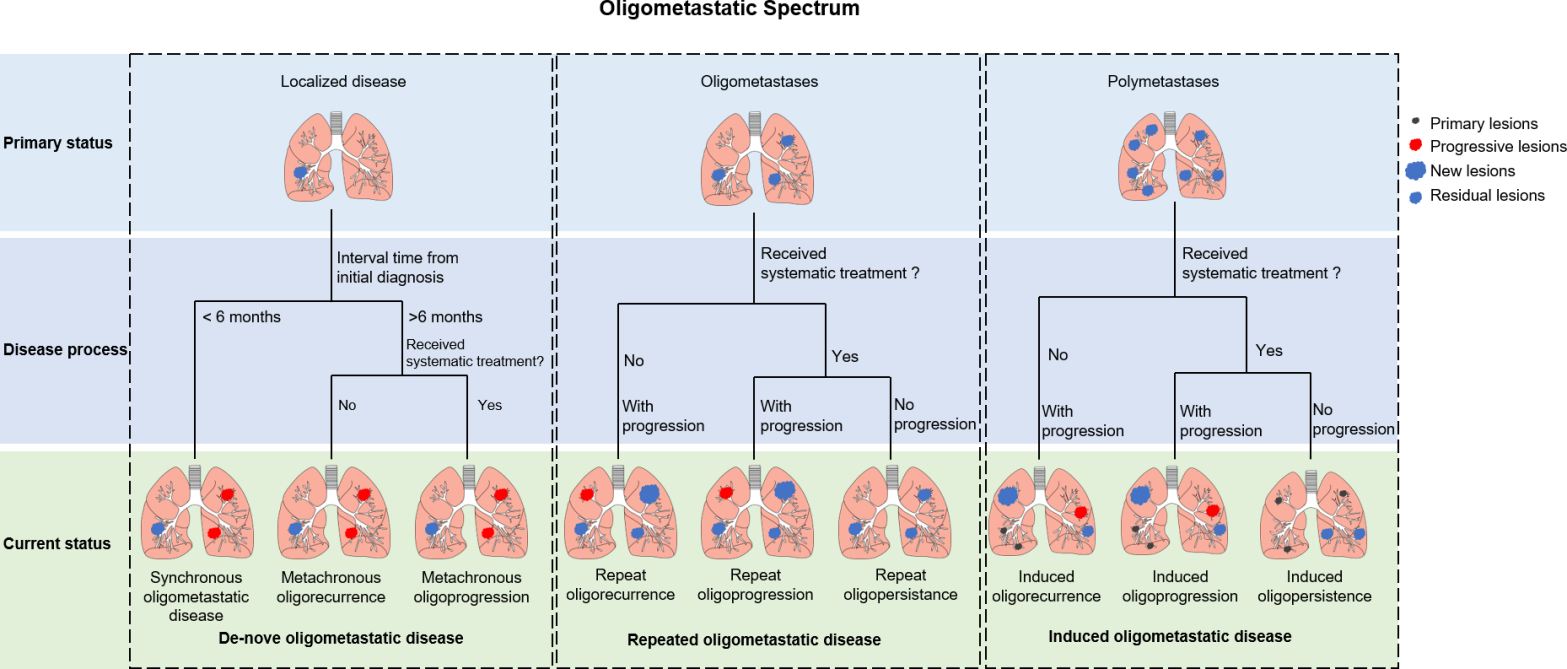
ESTRO/EORTC classification of oligometastatic disease.

OMD’s 5-year overall survival (OS) ranges between 8.3% to 86% (10), suggesting that OMD with similar imaging features may possess substantially different biological characteristics. Hellman and Weichselbaum proposed that oligometastases may arise from the progression of primary tumor or the eradication of widespread metastases (11). The ESTRO and EORTC have established a classification system to accurately describe the status of OMD, which can be categorized into three subtypes based on the patient’s historical diagnosis: *de-novo*, repeated, and induced OMD (**Figure 1**) (12). *De-novo* OMD occurs when patients do not have a prior history of metastases. It can be categorized as synchronous (< 6 months) or metachronous (> 6 months) based on the interval between primary and current diagnosis. Metachronous OMD can occur during a treatment-free interval (metachronous oligorecurrence) or during active systemic therapy (metachronous oligoprogression). Whereas repeat OMD represents OMD in patients with *de-novo* OMD that response to local and systemic treatments poorly; a state of OMD may be induced by systemic therapy in patients with polymetastatic disease. Repeat and induced OMD can also be classified into oligorecurrence (no treatment and progression), oligoprogression (treatment and progression), and oligopersistence (treatment but no progression), depending on the patient’s clinical status upon presentation.

## Immune checkpoint inhibitors improve the survival of stage IV NSCLC patients

In recent years, ICIs have significantly transformed the treatment of metastatic NSCLC. Monoclonal antibodies target specific inhibitory immune checkpoints, such as cytotoxic T-lymphocyte antigen-4 (CTLA-4), programmed death 1 (PD1), or programmed death ligand 1 (PD-L1), and have improved OS and progression-free survival (PFS) in patients with metastatic NSCLC in multiple large-scale phase III RCTs, particularly in patients with PD-L1 tumor proportion score (TPS) ≥ 50% (**Supplementary Table 1**). CheckMate-017 and CheckMate-057 evaluated Nivolumab as second-line treatment for advanced NSCLC, with improved PFS and OS compared to docetaxel (13, 14). In 2021, updated data showed that Nivolumab increased 5-year OS rate by over five-fold (13.4% vs. 2.6%) (15). Checkmate-078 further confirmed Nivolumab’s effectiveness in Chinese patients (16). However, Checkmate-026 did not demonstrate a survival benefit from Nivolumab over chemotherapy in the first-line setting (17). Alternatively, Checkmate-9LA and Checkmate-227 showed a benefit from first-line Nivolumab plus Ipilimumab in PD-L1 positive NSCLC patients (18, 19). Single-agent Pembrolizumab as a second-line treatment for metastatic NSCLC patients with PD-L1 TPS ≥ 1% provided a sustained and clinically meaningful improvement in PFS and OS, with more than doubled 5-year OS rate compared to docetaxel (20). For advanced NSCLC patients with a PD-L1 TPS >50%, the KEYNOTE-024 trial showed that single-agent Pembrolizumab provided long-term survival benefits, enabling patients to avoid chemotherapy (21). The KEYNOTE-042 trial further expanded the patient population benefiting from Pembrolizumab to include those with a PD-L1 TPS score ≥1% (22). For PD-L1 negative patients, Pembrolizumab plus chemotherapy has been established as a first-line treatment option based on the results from the KEYNOTE-189 and KEYNOTE-407 trials, which nearly doubled 5-year OS rate compared to chemotherapy alone (23, 24).

Atezolizumab, the first FDA-approved PD-L1 monoclonal antibody, is promising for advanced NSCLC. In the OAK trial, it improved OS compared to chemotherapy for previously treated NSCLC (median OS: 13.8 vs. 9.6 months, *p* = 0.0003) (25). In the first-line treatment of advanced NSCLC, the Impower110 trial showed that Atezolizumab monotherapy provided significant benefit over chemotherapy in patients with a high PD-L1 expression, offering another “chemotherapy waiver” treatment option (26). IMpower130 evaluated Atezolizumab plus chemotherapy for first-line treatment of non-squamous NSCLC patients with wild-type EGFR/ALK. PD-L1 expression was associated with better PFS (27). Impower150 added Atezolizumab to chemotherapy and Bevacizumab, significantly prolonging PFS and OS in metastatic non-squamous NSCLC patients, regardless of PD-L1 expression or EGFR/ALK status (28). Overall, Atezolizumab is promising for metastatic NSCLC patients, particularly those with high PD-L1 expression. Several other PD-1/PD-L1 inhibitors have also been safely applied in the first- and second-line treatment of advanced NSCLC (29–40). Despite the rapid clinical adaptation of ICI’s, most patients respond to ICI-containing regimens poorly. No consensus has been reached regarding to patient selection despite the exploration of over 40 potential predictive biomarkers for tumor response to ICIs (41). PD-L1 expression is the most accepted predictive biomarker for ICI response, but ≤ 26% of patients have PD-L1 levels above 50% (18). As a strategy to enhance treatment response in stage IV NSCLC, combining ICIs with local radiotherapy may be a promising approach to further enhance local and systemic tumor control (42).

## Local ablative therapies for oligometastatic NSCLC in the pre-ICI era

Local therapies like radiotherapy and surgery may cure some NSCLC patients with OMD (43). Comprehensive local therapy improved OS (27.1 vs. 13.1 months) and PFS (11.3 vs. 8.0 months) in NSCLC patients with ≤3 synchronous metastases (44). In a phase II study, local consolidative therapy (surgery, radiation therapy, or both) extended PFS from 4.4 to 14.2 months in 49 oligometastatic NSCLC patients (≤3 metastases) after first-line systemic therapy (45). In a single-arm phase II trial, a small number of 5-year survivors (7.7%) was observed after radical local treatment in oligometastatic NSCLC patients who also received systemic therapy (46). Furthermore, Xu et al. found that local ablative therapy may improve the survival of EGFR-mutant oligometastatic NSCLC patients treated with first-line EGFR-tyrosine kinase inhibitors (TKIs) (47). In the SINDAS trial, upfront radiotherapy added to first-line EGFR-TKI significantly improved PFS and OS in EGFR-mutated NSCLC patients with synchronous OMD (median PFS: 20.2 vs. 12.5 months; median OS: 25.5 vs. 17.4 months) (48).

SBRT is a safe and effective treatment for not only early stage, but also oligometastatic NSCLC, which represent a significant portion of patients with metastatic NSCLC (49–56). In several phase II trials, SBRT following chemotherapy or targeted therapy has been shown to improve survival outcomes in oligometastatic NSCLC patients (57–59). Ongoing RCTs are exploring whether SBRT improves survival in patients receiving maintenance therapy (60, 61). For second-line treatment of oligometastatic NSCLC, combining SBRT with targeted agents led to a median PFS of 14.7 months and a median OS of 20.4 months, which warrants further exploration (62). Overall, incorporating SBRT into the treatment regimen of oligometastatic NSCLC has been shown to be tolerable, and may lead to promising survival outcome. **Table 1** summarizes the prospective studies on SBRT for oligometastatic NSCLC.

**Table 1:** Clinical trials of SBRT in oligometastatic non-small-cell lung cancer.

Study	Study design	Inventions (n)	Outcomes
Gomez et al. (45)	RCT, phase II	Maintenance systemic therapy with vs. without local therapy (radiotherapy or surgery), after induction chemotherapy (n=49, with 1-3 metastases)	median PFS: 14.2 months vs. 4.4 months, P=0.022 median OS: 41.2 months vs. 17.0 months, P=0.017
De Ruysscher et al. (46)	Single-arm, phase II	Radical local treatment (surgery or radiotherapy) with chemotherapy or as a primary treatment (n=40, with ≤5 oligometastases)	median OS: 13.5 months
Wang et al. (48)	RCT, phase III	First-generation EGFR inhibitors with vs without upfront radiotherapy to all sites (n=133, with ≤5 metastases, ≤2 in any one organ and without brain metastases)	median PFS: 20.2 months vs. 12.5 months median OS: 25.5 months vs 17.4 months
Collen et al. (57)	Single-arm, phase II	SBRT was delivered after induction chemotherapy or as a primary treatment (n=26, with ≤5 metastases)	median PFS: 11.2 months
Iyengar et al. (58)	RCT, phase II	Maintenance systemic therapy with vs. without SBRT to all disease sites (primary plus 1-5 metastases), after induction chemotherapy (n=29)	median PFS: 9.7 months vs. 3.5 months, P=0.01
Palma et al. (59)	RCT, phase II	Palliative standard of care treatments with vs. without SABR to all metastatic lesions (n=99, with ≤5 metastases)	median OS: 41 months vs. 28 months
Iyengar et al. (62)	Single-arm, phase II	Erlotinib and SBRT to all sites of disease after failed early systemic chemotherapy (n=24, extracranial disease ≤6)	median PFS: 14.7 months median OS: 20.4 months

n, patient number; RCT, randomized controlled trials; PFS, progression-free surival; OS, overall survival.

## Clinical efficacy of SBRT combined with immunotherapy in metastatic NSCLC patients

Radiotherapy promotes the release of tumor-associated antigens and immune cell infiltration into the tumor, which can lead to an abscopal effect where unirradiated tumors respond (63). Combining SBRT with immunotherapy has gained great attention due to its ability to induce the abscopal effect (64). Compared with conventionally fractionated radiation therapy (CFRT), SBRT is associated with less lymphopenia and better clinical efficacy (65–67). Therefore, combining SBRT and immunotherapy appears promising as an emerging cancer treatment. The efficacy of combined radio-immunotherapy is affected by various factors, including radiation dose, fractionation schedule, treatment volume, and delivery sequence with an ICI. The most optimal dose fractionation schedule for maximum immune activation is not yet determined. In preclinical studies, either a single fraction of 15 Gy or 3 Gy×5 fractions elicited an immune response, but 15Gy x 1 fraction had a stronger immune-stimulatory effect (68). However, 7.5 Gy×2F and 5 Gy×3F were suggested to lead to superior anti-tumor immunity than a single ablative dose of 15 Gy. The medium-sized radiation dose of 7.5 Gy/F was recommended for best tumor control, due to its associated with strong anti-tumor immunity and low Treg numbers (69).

Although radiotherapy schedules varied in various trials, 8 Gy × 3 fractions has been very commonly used (70–72). Some studies found differences in efficacy to be associated with different dose fractionation regimens and treatment to different sites (73). Welsh et al. found that combining SBRT (50 Gy/4F) with immunotherapy yielded a 38% abscopal response rate (ARR), while the rate was only 10% with more protracted course of radiotherapy (45 Gy/15F) (74). Similar results were found in a pooled analysis of the MDACC and PEMBRO-RT trials favoring SBRT delivered in 3-4 fractions (67). Large-scale clinical studies are needed to explore the best SBRT dose and fractionation schedules to be combined with immunotherapy. Tumor regression in lesions exposed to low-dose irradiation during SBRT treatment has also been reported, suggesting the additional benefit of adding low-dose radiotherapy (LDRT) to selected metastatic lesions in patients receiving SBRT and immunotherapy (75, 76). Clinical trials investigating how to best combined LDRT, SBRT, and immunotherapy are warranted. Intra-tumoral heterogeneity may partially account for inconsistent efficacy of combining immunotherapy with SBRT (77).

Triggering of the abscopal effect is also by the tumor site(s) selected for local therapy, as not all metastatic sites are equally involved in systemic immune surveillance (78). For instance, irradiation of liver sites instead of lung sites may produce greater T-cell activation (79). However, since infiltration of activated T cells among multiple metastatic sites is complex and not intuitively predictable, SBRT to multiple or all tumor sites may be most effective for the generation of systemic antitumor immunity (67, 78, 80–82). This treatment approach is further supported by the following facts: acquired resistance to PD-1 axis inhibitors is often limited to no more than three metastatic sites (83); mixed progression is common in NSCLC (45%) and is associated with improved survival compared to those who experience widespread progression (84); SBRT to more than one lesion was shown to be associated with a trend toward improved overall tumor control (85).

Possible sequences of combing radiotherapy with immunotherapy include its administration before, concurrently with, or after radiotherapy. In preclinical studies, concurrent anti-PD-L1 agent and radiotherapy generated the most effective anti-tumor immune response (86). Buchwald et al. suggested that anti-PD-1/L1 and radiotherapy should be given simultaneously or, if not, radiotherapy should precede checkpoint blockers, as radiation to tumors after anti-PD-1/L1 therapy may abrogate the recently infiltrated and reactivated T-cell response (87). It is noteworthy that the above findings are primarily based on conventional fractionated radiotherapy, but for SBRT, intervention at any stage of immunotherapy may lead to survival benefits. Bestvina et al. presented the first randomized comparison of concurrent versus sequential dual checkpoint blockade and SBRT. They demonstrated no statistically significant difference in the median PFS between the concurrent and sequential groups (7.9 months vs. 4.7 months, *p* = 0.43) (73). Several clinical studies have also shown that the incidence of treatment-related toxicity following immunotherapy administered concurrently or sequentially with SBRT are not significantly different (70, 72, 73, 79). However, the current studies are largely limited by sample size, and the most optimal sequence of SBRT and immunotherapy needs further assessment in larger prospective trials.

Given the redundancy in the mechanisms of ICI- and radiation-induced toxic effects, radiation oncologists commonly avoid concurrent SBRT and ICI due to concerns about increased toxicity (88). However, current evidence only demonstrated a slightly elevated risk of certain toxic reactions in patients undergoing SBRT during immunotherapy (89). In the PEMBRO-RT trial, the addition of SBRT to Pembrolizumab increased the incidence of pneumonia from 8% to 26% and G3+ immune-related pneumonitis from 0% to 5%, but the total number of adverse events did not significantly differ from Pembrolizumab monotherapy (85 vs. 68 events, *p* = 0.076) (70). Multiple other studies have also demonstrated that the addition of SBRT to immunotherapy does not increase the incidence of severe adverse events (AEs) in patients with advanced NSCLC (71, 72, 74, 90). As shown in a large retrospective study, patients receiving radiotherapy within 90 days of starting ICI therapy may have a slightly higher incidence of adverse events (AEs), particularly low-grade AEs, compared to those receiving RT more than 90 days after starting ICI therapy (89). However, the clinical applicability of this conclusion to patients with advanced NSCLC still requires further verification given the varying AE spectra observed in different cancer types treated with PD-1 inhibitors (91). The sequence of SBRT and immunotherapy does not appear to induce significant difference in the incidence of severe AEs. Also shown by Bestvina et al. no statistical difference was present between patients receiving concurrent or sequential SBRT with the administration of Ipilimumab and Nivolumab (13 vs. 14 patients with G3+ immune-related AEs) (73). Other studies also confirmed the tolerability of concurrent SBRT and immunotherapy in NSCLC patients (72, 74, 79, 90). Welsh et al. found that the incidence of G3+ AEs was similar when either conventional fractionated radiotherapy or SBRT was combined with immunotherapy (74). Similarly, Schoenfeld et al. demonstrated no significant difference in the incidence of G3+AEs between the Durvalumab/Tremelimumab (D/T) plus LDRT group and the D/T plus SBRT group (15% and 12%, separately, P=0.27) (72). Reaching a consensus on the toxicity profile of the SBRT & ICI combination is difficult due to sample-size limitations and heterogeneities in patient population and treatment regimen. Meanwhile, the development of multisystem irAEs may also be associated with improved survival in patients with advanced NSCLC treated with ICIs (89, 92). Prospective trials are needed to investigate the most optimal sequence of combining radiotherapy and ICI to improve patient survival while maintaining a low incidence of treatment-related toxicities. It is also important to further explore how to best select patients for the combined treatment regimens. For example, some studies suggested that the combination of SBRT and immunotherapy may be more suitable for patients with low PD-L1 expression due to the better efficacy of immunotherapy alone in patients with high PD-L1 expression (70, 74). Thus, the selection of patients based on the presence and/or the level of specific biomarkers may help overcome the obstacle of hard-to-meet study endpoints in current clinical trials. Further investigations in this area are warranted. A summary of the clinical outcome and toxicity profile following SBRT combined with ICIs in prospective studies is provided in **Table 2**.

**Table 2:** Clinical trial summary of SBRT and immunotherapy combination therapy for advanced non-small-cell lung cancer.

Study	Design	Inventions	Fractionation	Target volume	Sequence	Outcomes	Toxicity
Theelen et al. (70)	RCT, phase 2	Pembro after vs. without radiotherapy (n=92, failed first-line chemotherapy)	24Gy/3F	Partial (1 lesion)	SBRT before immunotherapy	mPFS:1.9 vs. 6.6 months (P=0.19) mOS: 7.6 vs. 15.9 months (p=0.16)	Pneumonia (8% vs. 26%, P=0.06); G3+ immune-related pneumonitis (0% vs. 5%)
Ni et al. (71)	Single-arm, phase 2	SBRT followed by Sintilimab and GM-CSF (n=20, failed first-line chemotherapy)	24Gy/3F	Partial (1 lesion)	SBRT before immunotherapy	The triple regimen is safe and well tolerated	No patients had DLTs and 18 patients experienced treatment-related AE.
Schoenfeld et al. (72)	RCT, phase 2	Durvalumab/Tremelimumab alone vs. with LDRT vs. with HFRT(n=90)	LDRT: 0.5 Gy bid x 8 days; HFRT: 8Gy×3F	Partial (1-2 lesions)	Concurrent	ORR: 11.5% vs. 7.7% vs. 11.5%; DCR: 30.8% vs. 23.1% vs. 34.6%	G3+ AE possibly related to study therapy (15% vs. 31% vs. 12%, P=0.27); median follow-up time was 12.4 months
Bestvina et al. (73)	RCT, phase 1	Concurrent vs. sequential SBRT with Nivolumab and Ipilimumab (n=37)	30Gy/3F; 45Gy/3F; 50Gy/5F	Partial (2-4 lesions)	Concurrent or sequential	mPFS: 7.9 vs. 4.7 months (P= 0.43)	G3+ irAEs in 13 patients in the concurrent group vs. 14 patients in the sequential group; median follow-up time was 17 months
Welsh et al. (74)	RCT, phase 1/2	Pembro vs. Pembro+SBRT vs. Pembro+CFRT (n=100, with 1-4 lung or liver lesions)	SBRT: 50Gy/4F; CFRT: 45Gy/15F	Partial	Concurrent	Out-of-field ORR: 25% vs. 38% vs. 10%; PFS: 5.1 vs. 20.8 vs. 6.8 months	Two G4 AE and two G3 AE in the Pembro+SBRT group; Five G3 AE in Pembro+ CFRT group; median follow-up time was 20.4 months
Mattes et al. (91)	Single-arm	ICI with vs. without SBRT (n=35, CPI-naïve)	48Gy (IQR:43 – 60 Gy)/3–5 F	Partial	Concurrent	mOS: 15.0 months ; mPFS: 6.9 months; mTTP: 11.2 months	Radiation-induced toxcity (56% vs. 32%, P<0.01), no G3+ radiation-induced toxicities; median follow-up time was 14.0 months

RCT, random clinical trial; mOS, median overall survival; mPFS, median progression-free survival; SBRT, stereotactic body radiotherapy; DLT, dose-limiting toxicity; AE, adverse event; irAE, immune-related adverse event; LDRT, low-dose radiation; HFRT, hypofractionated radiation; CFRT, conventional fractionated radiation; ORR, overall response rate; DCR, disease control rate.

## Conclusion

Combining SBRT with immunotherapy is a promising treatment option for patients with metastatic NSCLC, especially those with OMD. Despite the emergence of early clinical evidence, the most optimal strategy for combining SBRT and immunotherapy remains to be further investigated to determine the most effective radiation dose/fractionation, target, and sequence with immunotherapy. For instance, the 8 Gy x 3 fractions regimen led to more prominent abscopal effect than higher single fractional dose of 20 Gy *in vivo*; likely due to DNA exonuclease Trex1 induction by high fractional doses above 12-18 Gy, leading to subsequent cytosolic DNA degradation and decreased immunogenicity (93, 94). Such finding led to the question of whether doses that are much lower than a biologically effective dose (BED) of 100 Gy_10_ should be considered when delivering “immunogenic” SBRT in combination with ICIs, in contrast to delivering ablative doses with a BED of ≥ 100 Gy_10_. Full dose SBRT remains to be recommended when it’s combined with ICIs for metastases based on limited evidence and expert opinion by the EORTC-ESTRO OligoCare consortium (95). In the evidence reviewed by this consortium, the risk of in-field severe toxicity was increased when thoracic SBRT was combined with an anti-CTLA-4 antibody (12%), or anti-PD-(L)1/anti-CTLA-4 combinations (26%). The majority of experts in the consortium recommended not to deliver an ICI and SBRT on the same day, and a time interval of at least one week between anti-CTLA-4 or anti-CTLA-4/anti-PD-(L)1 antibodies and SBRT administration (95). Given the increased utilization of local therapy with systemic therapy in the management of oligometastatic NSCLC, a clinical practice guideline was also released jointly by the ASTRO and ESTRO in 2023 (96). In this guideline, oligometastatic NSCLC’s definition, choice of local treatment and its sequencing with systemic therapy, radiotherapy dose/fractionation and techniques, as while as the indications for additional local therapy upon progression were reviewed. It further defined durable local control as a minimum of 85% at 2 years, while recognizing the likelihood of acceptable local control following SBRT delivering lower BED’s (50-75 Gy_10_) when it’s combined with systemic therapy. As implied by the European and European/American consensus recommendations, additional efforts should be directed toward conducting high quality clinical investigations into how to effectively combine SBRT and immunotherapy, which will provide insights and ultimately improve the care of patients with advanced stage NSCLC.

## Author contributions

XL drafted the manuscript, created the figures and tables. AC generated the review’s concepts and structure. AC completed the final editing of the manuscript. All authors contributed to the article and approved the submitted version.
