# Supplementary material for: Combining stereotactic body radiotherapy with immunotherapy in stage IV non-small cell lung cancer
Source: Front Oncol. 2023 Sep 6;13:1211815. doi: 10.3389/fonc.2023.1211815 (PMC10511897; doi:10.3389/fonc.2023.1211815)
Supplement: Supplementary file 1 [file Table_1.docx]

**Supplementary Table 1.** Phase Ⅲ randomized controlled trials of immune checkpoint inhibitors for stage IV non-small cell lung cancer

| **No.** | **Study** | **Line of treatment** | **N** | **Intervention** | **Outcomes** |
| --- | --- | --- | --- | --- | --- |
| 1 | CheckMate-017 (13) | 2nd/3rd | 272 | Nivolumab vs. Docetaxel | mOS: 9.2 vs. 6.0 months, P<0.001  mPFS: 3.5 vs. 2.8 months, P<0.001 |
| 2 | CheckMate-057 (14) | 2nd/3rd | 582 | Nivolumab vs. Docetaxel | mOS: 12.2 vs. 9.4 months, P=0.002  mPFS: 2.3 vs. 4.2 months, P=0.39 |
| 3 | CheckMate-078 (16) | 2nd/3rd | 451 | Nivolumab vs. Docetaxel | mOS: 12.0 vs. 9.6 months, P=0.0006 |
| 4 | CheckMate-026 (17) | 1st | 423 | Nivolumab vs. Platinum-based chemotherapy | mOS: 14.4 vs. 13.2 months  mPFS: 4.2 vs. 5.9 months, P=0.25 |
| 5 | Checkmate-9LA (18) | 1st | 719 | Nivolumab + Ipilimumab + chemotherapy vs. chemotherapy | mOS: 15.6 vs. 10.9 months |
| 6 | CheckMate-227 (19) | 1st | 1,739 | Nivolumab + Ipilimumab vs. Platinum-based chemotherapy | mOS: 17.1 vs. 14.9 months (PD-L1 TPS≥1%); 17.4 vs. 12.2 months PD-L1 TPS＜1%)  mPFS: 5.1 vs. 4.2 months (PD-L1 TPS≥1%); 5.1 vs. 5.6 months (PD-L1 TPS＜1%) |
| 7 | Keynote-010 (20) | 1st | 1,034 | Pembrolizumab vs. Docetaxel | mOS: 16.9 vs. 8.2 months (PD-L1 TPS≥50%); 11.8 vs. 8.4 months (PD-L1 TPS≥1%)  mPFS: 5.3 vs. 4.2 months (PD-L1 TPS≥50%); 4.0 vs. 4.1 months PD-L1 TPS≥1%) |
| 8 | Keynote-024 (21) | 1st | 305 | Pembrolizumab vs. Platinum-based chemotherapy | mOS: 30.3 vs. 14.2 months, P=0.002 |

**Supplementary Table 1.** Phase Ⅲ randomized controlled trials of immune checkpoint inhibitors for stage IV non-small cell lung cancer (continued)

| **No.** | **Study** | **Line of treatment** | **N** | **Intervention** | **Outcomes** |
| --- | --- | --- | --- | --- | --- |
| 9 | Keynote-042 (22) | 1st | 1,274 | Pembrolizumab vs. Platinum-based chemotherapy | mOS: 20 vs. 12.2 months (PD-L1 TPS≥50%, P=0.0003); 17.7 vs. 13.0 months (PD-L1 TPS≥20%, P=0.0020); 20 vs. 12.2 months (PD-L1 TPS≥1%, P=0.0018); |
| 10 | Keynote-189 (23) | 1st | 616 | Pembrolizumab + Platinum-based chemotherapy vs. chemotherapy | mOS: 22 vs. 10.6 months  mPFS: 9 vs. 4.9 months |
| 11 | Keynote-407 (24) | 1st | 559 | Pembrolizumab + chemotherapy vs. chemotherapy | mOS: 17.1 vs. 11.6 months,  mPFS: 8 vs. 5.1 months, |
| 12 | OAK (25) | 2nd/3rd | 1,225 | Atezolizumab vs. Docetaxel | mOS: 13.8 vs. 9.6 months, P=0.0003 |
| 13 | IMpower110 (26) | 1st | 554 | Atezolizumab vs. Platinum-based chemotherapy | mOS: 20.2 vs. 14.7 months (PD-L1 TPS ≥50% or CPS ≥10%) |
| 14 | IMpower130 (27) | 1st | 724 | Atezolizumab + Carboplatin + Nab-paclitaxel vs. Carboplatin + Nab-paclitaxel | mPFS:7.0 vs. 5.5 months, P < 0.0001  mOS: 18.5 vs. 13.9 months, P=0.033 |
| 15 | IMpower150 (28) | 1st | 1,202 | Atezolizumab + Bevacizumab + Carboplatin + Paclitaxel (ABCP) vs. Bevacizumab + Carboplatin + Paclitaxel (BCP) | mOS: 19.8 vs. 15.0 months  mPFS: 8.4 vs. 6.8 months |
| 16 | ORIENT-11 (29) | 1st | 397 | Sintilimab + chemotherapy vs. chemotherapy | mPFS: 9.2 vs. 5.0 months, P < 0.0001  mOS: NR vs. 16.8 months, P=0.0003 |
| 17 | ORIENT-12 (30) | 1st | 543 | Sintilimab + chemotherapy vs. chemotherapy | mPFS: 5.5 vs. 4.9 months, P<0.00001 |
| 18 | ORIENT-31 (31) | 1st | 936 | Sintilimab + IBI305 + chemotherapy vs. chemotherapy | mPFS: 6.9 vs. 4.3 months, P<0·0001 |

**Supplementary Table 1.** Phase Ⅲ randomized controlled trials of immune checkpoint inhibitors for stage IV non-small cell lung cancer (continued)

| **No.** | **Study** | **Line of treatment** | **N** | **Intervention** | **Outcomes** |
| --- | --- | --- | --- | --- | --- |
| 19 | RATIONALE 303 (32) | 2nd/3rd | 805 | Tislelizumab vs. Docetaxel | mOS: 16.9 vs. 11.9 months, P<0.0001 |
| 20 | RATIONALE 304 (33) | 1st | 332 | Tislelizumab + Platinum vs. Platinum | mPFS: 9.7 vs. 7.6 months, P= 0.0044 |
| 21 | RATIONALE 307 (34) | 1st | 355 | Tislelizumab + Paclitaxel + Carboplatin vs. Tislelizumab + Nab-paclitaxel + Carboplatin vs. Paclitaxel + Carboplatin | mPFS: 7.6 vs. 7.6 months vs. 5.5 months, P<0.001 |
| 22 | EMPOWER-Lung1 (35) | 1st | 710 | Cemiplimab vs. Platinum-doublet chemotherapy | mPFS: 8.2 vs. 5.7 months, P<0.0001  mOS: NR vs. 14,2 months, P=0·0002 |
| 23 | EMPOWER-Lung 3 (36) | 1st | 466 | Cemiplimab + Platinum-doublet chemotherapy vs. platinum-doublet chemotherapy | mOS: 21.9 vs. 13 months, P= 0.014  mPFS: 8.2 vs. 5.0 months, P < 0.0001 |
| 24 | CameL (37) | 1st | 419 | Camrelizumab + Pemetrexed vs. Pemetrexed | mPFS: 11.3 vs. 8.3 months, P=0·0001 |
| 25 | CameL-sq (38) | 1st | 389 | Camrelizumab + chemotherapy vs. chemotherapy | mPFS: 8.5 months vs. 4.9 months, P<0.0001  mOS: NR vs. 14.5 months, P<0.0001 |
| 26 | GEMSTONE-302 (39) | 1st | 479 | Sugemalimab + chemotherapy vs. chemotherapy | median PFS: 9.0 vs. 4.9 months, P < 0.0001 |
| 27 | CHOICE-01 (40) | 1st | 465 | Toripalimab + chemotherapy vs. chemotherapy | mPFS: 8.4 vs. 5.6 months, P<0.0001 |

mOS, median overall survival; mPFS, median progression-free survival; PD-L1, programmed cell death ligand 1; TPS, tumor proportion score; NR, not reached.
